# Compact Integrated On-Chip MIMO Antenna with Reconfigurability for mmWave Frequencies

**DOI:** 10.3390/s25041062

**Published:** 2025-02-10

**Authors:** Khaled Boubekeur, Nicolas Zerounian, Badr Eddine Ratni

**Affiliations:** 1 Centre de Nanosciences et de Nanotechnologies (C2N), CNRS, Université Paris-Saclay, F91120 Palaiseau, France; nicolas.zerounian@universite-paris-saclay.fr; 2 LEME, UPL, Université Paris Nanterre, F92410 Ville d’Avray, France

**Keywords:** on-chip antennas, MIMO, artificial magnetic conductor, BenzoCycloButene, metasurface, microelectronic processes

## Abstract

This paper presents a compact on-chip multiple-input multiple-output (MIMO) antenna designed for future communication systems, featuring frequency-agile elements. The antenna achieves enhanced decoupling and reduced cross-section through the integration of a metasurface, which also introduces frequency agility. Designed for the millimeter-wave band using low-loss BenzoCycloButene (BCB) polymer, the antenna is manufactured with microelectronic processes, and the dimensions are 7.54 × 7.54 × 0.055 mm^3^. Simulations and measurements demonstrate excellent frequency agility around 60 GHz, with gains of 6.5 to 9 dBi. As a proof of concept, open and short circuits were used for switching, with future designs aiming to incorporate diodes for a full dynamic reconfiguration. This work highlights the potential for compact, high-performance, and frequency-reconfigurable on-chip antennas in next-generation millimeter-wave systems.

## 1. Introduction

The rapid advancement of digital technology has driven a dramatic increase in data generation [1], requiring ever-higher data rates to enable efficient communication. Current wireless systems operating in frequency bands from MHz to tens of GHz have limited transmission capacity and may not meet future requirements. To address this challenge, emerging communication systems targeted higher frequency bands, such as 5G (around 30 GHz) and WiGig (Wi-Fi around 60 GHz), which offer greater transmission capacity [2,3,4,5]. The FCC has opened up the terahertz spectrum for potential 6G applications, promising significant data rates due to the wide channel bandwidths available at these frequencies [6].

However, the shift to higher frequencies brings challenges, particularly shorter wavelengths, which necessitate the miniaturization and integration of RF systems to minimize interconnection losses and mitigate the high atmospheric attenuation at these frequencies [7]. Today, the literature features numerous studies on highly integrated RF systems and circuits on chips, particularly in the mmWave and THz ranges, such as complete transceiver systems [8]. In this context, integrated antennas are indispensable for achieving compact and efficient wireless communication systems. Their design must simultaneously address multiple performance requirements, including wide bandwidth, reconfigurability, and advanced functionalities like diversity (e.g., MIMO systems) while being seamlessly integrated into transceiver chips [9,10,11].

In this article, we propose the design of an integrated antenna system for 60 GHz Wi-Fi. The system features MIMO antennas with frequency reconfiguration capabilities to effectively cover a wide frequency band. To meet these demanding requirements, metasurface-based antennas present a promising solution. Metasurfaces, derived from metamaterials, are engineered by arranging small scatterers or openings in a precise pattern on a 2D surface. Over the past decade, they have evolved from theoretical constructs to commercially viable technologies [12,13]. These structures have revolutionized antenna design by overcoming the limitations of traditional antennas, enabling enhanced directivity, reconfigurability, and low losses. They can therefore be leveraged to develop even more efficient integrated antenna systems [14].

In this work, we present an on-chip four-port MIMO antenna that integrates the benefits of metasurface-based isolation, compactness, and frequency agility, integrated onto BenzoCycloButene (BCB) as a substrate [15]. Available in resin for microelectronic fabrication processes, this polymer offers advantageous characteristics, including low losses (tg δ = 0.0008) and low dielectric constants (εr = 2.65) at frequencies in the tens of GHz, as well as at 1 THz (tg δ = 0.008 and ε_r_ = 2.45) [16]. These properties contribute to improving antenna efficiency and facilitate the extension of the design to the submillimeter-wave range, as already achieved for unimodal coplanar waveguides up to 760 GHz [17]. Herein, our design targets Wi-Fi (802.11ay) applications at 60 GHz [18]. By combining metasurface technology with BCB integration, our approach delivers an optimal balance between size, performance, and reconfigurability, positioning it as a strong candidate for the next generation of wireless communication systems.

## 2. Antenna Design and Simulations

**Figure 1:**
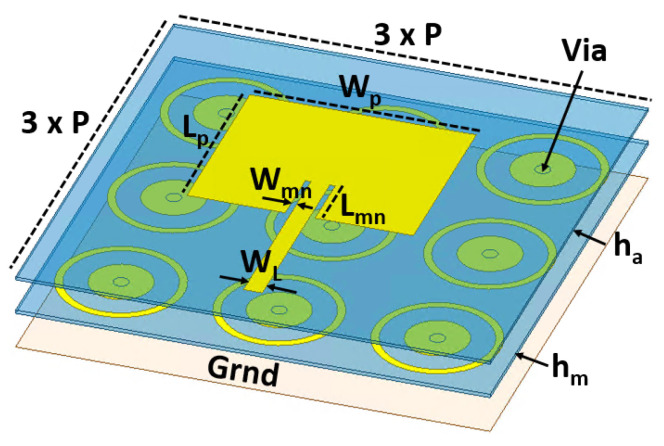
Schematic view of the proposed MIMO antenna structure based on a conventional patch antenna positioned above a metasurface.

**Figure 2:**
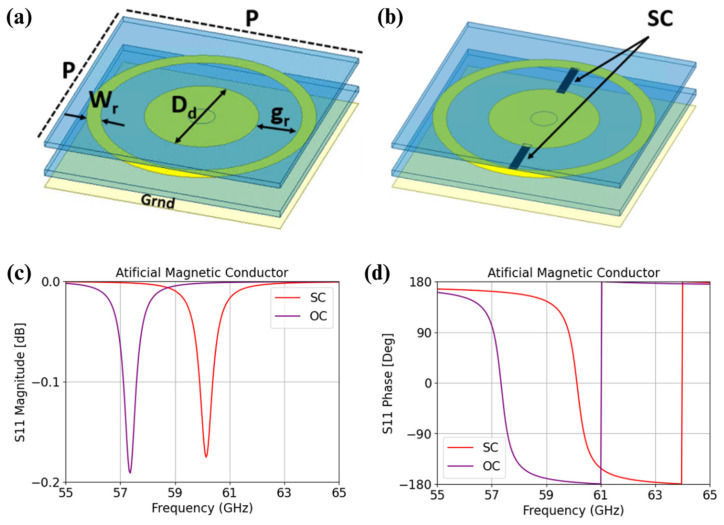
Schematic view of the metasurface unit cell (**a**) in OC configuration and (**b**) in SC configuration, with dielectric layers drawn with a separation between them and a simulation of the AMC behavior of the metasurface with (**c**) the magnitude of the reflected E field and (**d**) phase of the reflected E field at the patch level.

The idea is to design a compact MIMO antenna consisting of 4 × 4 elements which can be directly integrated onto an electronic chip for future communication systems. A frequency band around 60 GHz has been selected as the operating band for the development of this antenna, intended for future communication systems requiring, for example, two separate bands for uplinking and downlinking. The antenna is designed using microelectronic fabrication processes, with BenzoCycloButene (BCB) resin to cure the dielectric substrate and gold layer deposition for each metal level. BCB is chosen to minimize dielectric losses even at 60 GHz (εr = 2.65 and tg δ = 0.0008). BCB requires only 250 °C for a complete crosslinking compared to polyimide of a little higher permittivity with almost 350 °C. With a thickness ranging from few microns to few tens of microns, such a dielectric allows for monolithic IC designs of small dimensions matched to the sub-mm wavelength range compared to PCBs. For this purpose, the proposed MIMO antenna structure is based on a conventional linearly polarized patch antenna positioned above a metasurface, as shown in Figure 1. The metasurface serves two key functions. First, it enhances compactness by acting as an artificial magnetic conductor (AMC). This allows for a significant reduction in the total thickness of the substrate between the antenna and the ground plane to only 55 µm with the metasurface compared to the λ0/4 = 770 µm without a metasurface for the chosen polymer at 60 GHz with ε_r_ = 2.65. The metasurface achieves this by forcing the phase of the reflected E field to 0° at the patch level, as shown in Figure 2c,d. Secondly, the metasurface is within the frequency agility by the integration of active components in it. This frequency agility, in addition to broadening the operational bandwidth and enabling frequency reuse, enhances decoupling when the elements of the same MIMO antenna are detuned.

These two properties significantly improve the performance of the MIMO antenna. The key performance metrics of a MIMO system, including diversity parameters, are primarily determined by the gain and the level of coupling between the ports [19].

To ensure polarization insensitivity, each elementary cell of the metasurface is designed circularly. These cells are arranged in a square grid to maintain symmetry with the patch antenna, as depicted in Figure 1, and to the side-to-side symmetry of the MIMO antenna array described later. The elementary cells consist of metallic disks that are short-circuited at the center to the ground plane through a via. As shown in Figure 2, a metallic ring is incorporated around each disk to modulate the electromagnetic response. The frequency resonance agility is achieved by tuning the spacing or the coupling between the disk and the surrounding ring. This can be electronically controlled by adding diodes between the disk and the ring to alter the capacitive coupling without changing the physical dimensions. Each diode will exhibit capacitive coupling behavior, acting as an open circuit (OC) or a short circuit (SC) depending on the applied DC bias. Although the current prototype does not include diodes due to the challenges of integration and of biasing at this stage of development, their effects have been replicated.

In future prototypes including diodes, separated diodes can be picked and placed before the ground metal level for antenna, or diodes are fabricated in the carrier substrate. Prior to the ground plane for antennas, first levels ensure bias interconnection. Opening through the ground plane can allow some via to reach the metasurface. A design must be adapted to include such a way to connect the disk to the ring.

Instead of diodes, a 40 µm wide metal strip is used (two strips on each side aligned with the patch antennas), allowing the resonant frequency of the antenna to be effectively varied by simulating the OC (Figure 2a) or SC behavior (Figure 2b) of the diodes.

Simulations of metasurface unit cells and of the full array, followed by optimizations with the patch antennas, led to the final design, which had a disk diameter, D_d_, of 500 µm; a ring width, W_r_, of 62.7 µm; and a separation, g_r_, of 189.6 µm between the disk and the ring. The diameter via D_v_ is 120 µm. The metasurface’s periodicity is *p* with 1.256 mm. A manufacturing constraint is not to exceed 30 µm in thickness for each BCB layer.

**Figure 3:**
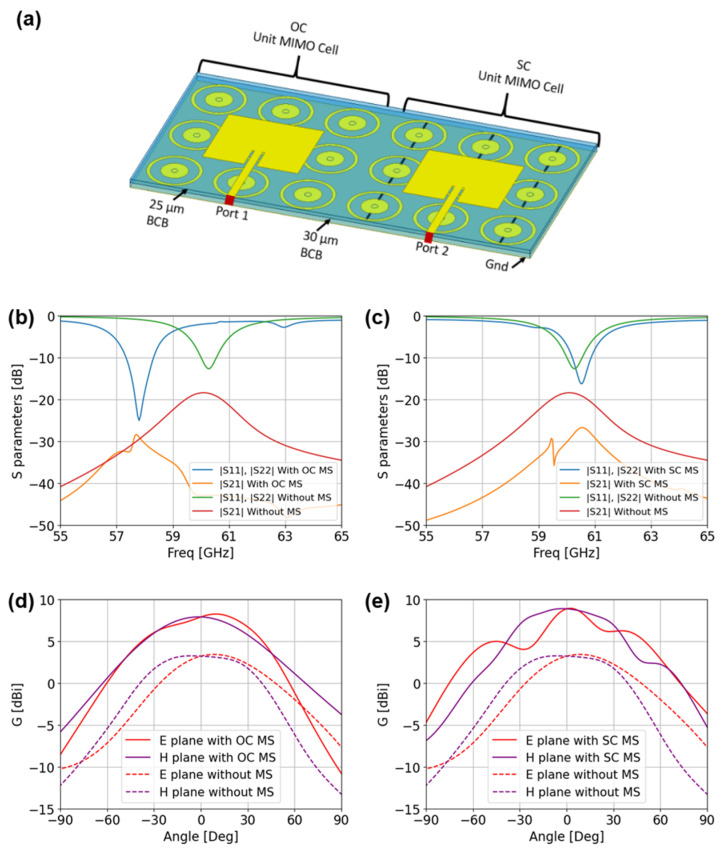
(**a**) Schematic view of the 2 × 2 MIMO antenna, showing the OC and SC configurations. (**b**) S-parameter comparison with and without the metasurface (MS) in the OC configuration. (**c**) S-parameter comparison with and without the MS in the SC configuration. (**d**) Simulated radiation patterns at 57.75 GHz for the OC configuration, with and without the MS. (**e**) Simulated radiation patterns at 60.5 GHz for the SC configuration, with and without the MS.

The patch antennas had dimensions of L_p_ = 1.8 mm × W_p_ = 1.5 mm. These were fed by 50 Ω microstrip lines 150 µm wide (W_L_), with impedance matching notches of L_mn_ = 0.48 mm × W_mn_ = 0.044 mm. A perspective view of two antennas is shown in Figure 3a. The metasurface was placed h_m_ = 30 µm above the ground plane, with a h_a_ = 25 µm BCB layer separating it from the patch antennas. The patch was surrounded by nine unit cells of the metasurface. In Figure 3a, the MIMO antenna on the left left is in the OC configuration, while the antenna on the right is in the SC configuration. Simulation of S-parameters between the two ports and radiation patterns are shown in Figure 3b,c.

We observed in Figure 3b that the antenna in the open-circuit configuration achieves a bandwidth of 1000 MHz, between 57.25 GHz and 58.25 GHz, with a gain of 7.2 dBi to 8.6 dBi and an efficiency of 90%. The antenna in a short-circuited configuration offers a bandwidth of 700 MHz, ranging from 60.2 GHz to 60.9 GHz. This configuration provides a gain that varies between 8.5 dBi and 9 dBi with an efficiency of 90%. The isolation seen with S21 is better than −35 dB. To highlight the effects of the metasurface, Figure 3 presents S-parameters and the realized gain of a 2 × 2 MIMO antenna with 55 µm of BCB without the metasurface (MS) and with the MS in its two configurations. The results clearly show that the coupling is significantly reduced by approximately −10 dB when the MS is introduced. Furthermore, the realized gain is enhanced by around 4 dB. These observations confirm the beneficial impact of the metasurface in mitigating coupling and improving the radiation performance of the antenna system without requiring additional spacing between antennas to reduce coupling or increased thickness to enhance the gain. Such enhancements demonstrate the potential of the metasurface as a valuable design element for optimizing antenna characteristics, particularly in scenarios where high isolation and improved gain are critical.

## 3. Diversity Parameters

**Figure 4:**
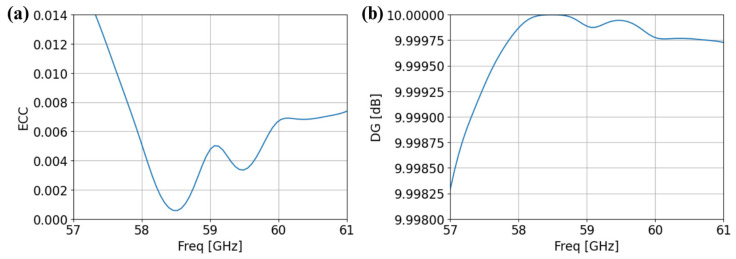
Diversity parameters in OC and SC configurations (**a**) for ECC and (**b**) for DG.

When dealing with MIMO antennas, it is essential to evaluate their diversity parameters, particularly the envelope correlation coefficient (ECC) and diversity gain (DG) [20]. ECC measures the correlation between MIMO antenna ports and is a critical parameter in determining the level of isolation and independence between antenna elements. It can be calculated from the radiation patterns or S-parameters [20]. A low ECC value indicates reduced coupling and improved performance, as it ensures the signals received or transmitted by the MIMO elements are largely uncorrelated, and the ECC should be less than 0.5 [20]. Diversity gain, on the other hand, quantifies the improvement in signal reliability provided by multiple antennas under fading conditions, directly impacting the MIMO system’s effectiveness in multipath environments. The DG should be equal to 10 dB [20]. The ECC and DG of the 2 × 2 MIMO antenna depicted in Figure 3a are shown in Figure 4a,b, respectively. The ECC is close to 0 and DG is close to 10 dB. The values of the ECC and DG are in the acceptable range and hence, the proposed design is suitable for different MIMO applications.

## 4. Fabrication and Experimental Validation

**Figure 5:**
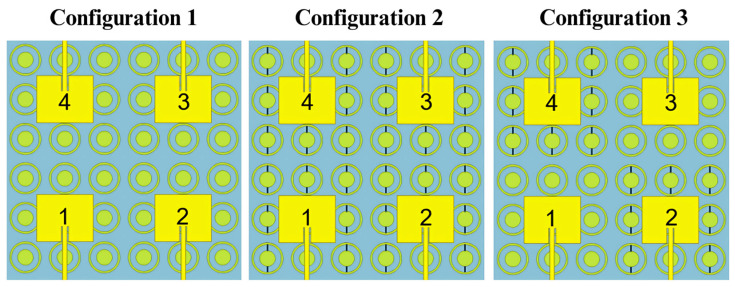
Schematic view of the 4 × 4 MIMO antenna, illustrating three OC and SC configurations. In Configuration 1, all four antennas are open circuit (OC); in Configuration 2, they are all short circuit (SC); and in Configuration 3, antennas 1 and 3 are OC, while antennas 2 and 4 are SC. Small black lines indicate short circuits.

Using these OC and SC metasurface configurations, three different 4 × 4 MIMO structures were designed as shown in Figure 5 and fabricated to serve as proofs of concept for reconfigurability.

**Figure 6:**
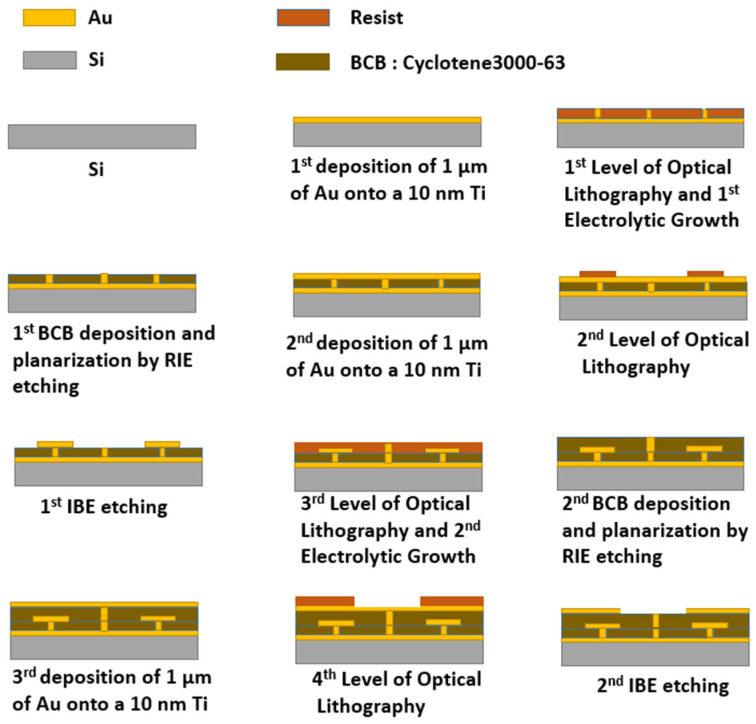
Illustration of the sample fabrication steps.

The fabrication of the sample is achieved with a microelectronic manufacturing process. The manufacturing steps, with four UV lithographic levels, are illustrated in Figure 6. First, starting from a 500 µm thick two-inch silicon wafer, which serves as a carrier substrate, the ground plane is formed with a 1 µm thick Au layer, deposited by electron beam evaporation, with a 10 nm thick Ti adhesion layer. Next, interconnecting vias between the ground plane and the metal layer defining the metasurface are obtained by electrolytic growth through a 40 µm thick UV-sensitive resist layer (AZ40XT). After the growth of vias a little higher than 30 µm, the resist is replaced by BCB (Cyclotene3000-63 from DuPont, Wilmington, DE, USA) with a thickness of 30 µm and cured at 75%. A reactive-ion etching of BCB is performed through a photoresist mask to remove the residual BCB on top of the vias. This is the same mask used for the vias. A second 1 µm gold layer is deposited on the first BCB layer, on which a second electrolytic growth is performed for interconnecting vias up to the last third metal layer on top of the stack. Once the second level of vias is completed and the photoresist removed, the gold layer on top of the first BCB layer is etched by ion beam etching (Ar) to form the disks and rings of the metasurface. A second layer 25 µm thick of BCB is deposited and cured with the same parameters as the first BCB layer. It follows the etching of BCB on top of the vias, and a final 1 µm thick gold layer is deposited and etched to form the patch antennas, the microstrip lines, and the coplanar access pads for measurement with GSG probes.

**Figure 7:**
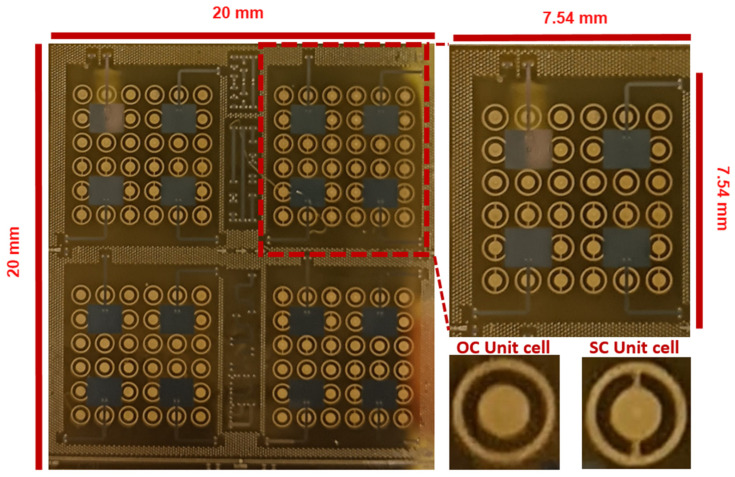
Picture of the fabricated prototype showing four zones with different 4 × 4 MIMO configurations, one 4 × 4 MIMO antenna magnified view, and open circuit unit cell and short-circuited unit cell magnified view.

Figure 7 shows the picture of the sample around the four combinations of MIMO antennas. For GSG probe measurement motivations with two independent GSG probes face-to-face or with a 90° orientation between them, 90° microstrip bends of the fed lines are used for some of the antennas. Each fed line includes the coplanar-to-microstrip transition. Some lines and reflects are included in the middle of the sample for calibration or de-embedding purposes.

**Figure 8:**
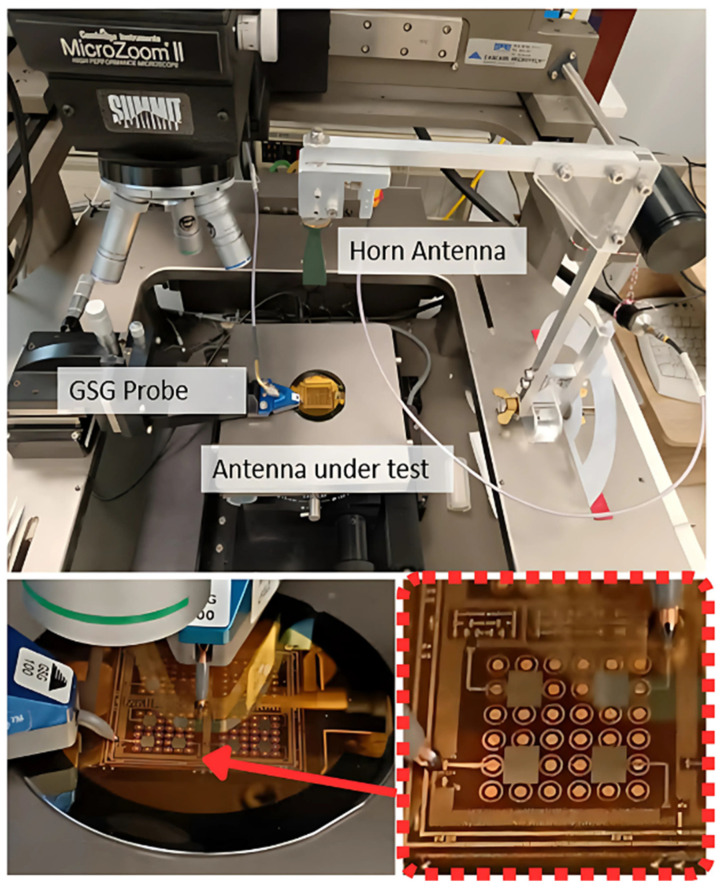
Fabricated sample and measurement setups of the scattering parameters and radiation patterns.

To experimentally validate the fabricated structure, S-parameter and radiation pattern measurements are conducted using the setup illustrated in Figure 8. Around a probe station, this setup includes a two-port Keysight E8361C vector network analyzer (manufactured by Keysight Technologies, located in Santa Rosa, CA, USA), two GSG probes (100 µm pitch) for measuring reflection and coupling coefficients from 10 MHz to 67.5 GHz (uncalibrated up to 70 GHz), and a rectangular horn antenna (20 dB gain, WR-15 to coaxial 1.85 mm adapter) on a motorized arm for radiation pattern measurements. TRL calibration patterns are also manufactured in the middle of the sample (Figure 7).

Measuring at 60 GHz presents challenges such as probe alignment sensitivity, calibration accuracy, and increased signal losses. The precise positioning of the GSG probes is crucial to ensure consistent contact, as even small misalignments can impact results. Additionally, the high atmospheric attenuation at 60 GHz requires careful control of the measurement distance to minimize losses while ensuring far-field conditions. Proper calibration and de-embedding are essential to obtain accurate measurements of the antenna’s performance. A SOLT calibration is performed at the GSG probe level, ensuring good measurement for reflection and coupling coefficients. For radiation measurement, only relative transmission coefficients are obtained from planar antennas. The horn is connected to port 2 of the VNA, while the calibrated port 1 feeds the antenna with the same GSG probe. It should be noted that the metallic sample holder of the probe station acts as an infinitively wide ground plane. Antennas are on their two-inch-wide ground plane, which limits effective angular range.

## 5. Measurements Results

**Figure 9:**
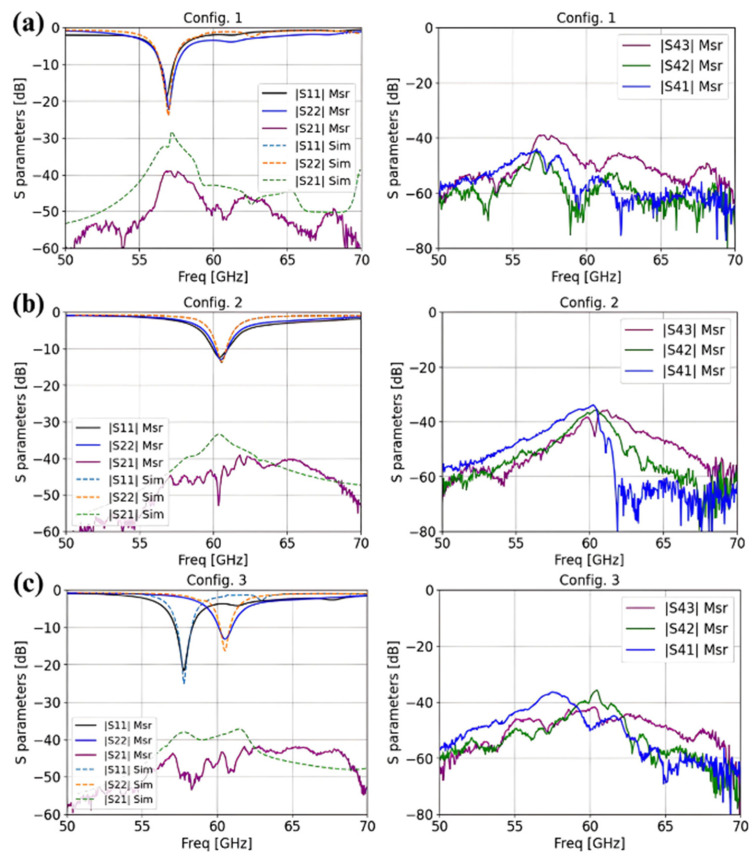
Measured and simulated reflection coefficients and coupling parameters for (**a**) configuration 1, (**b**) configuration 2, and (**c**) configuration 3.

The measured and simulated S-parameters for these three structures are shown in Figure 9a–c, corresponding to configurations 1, 2, and 3, respectively. The antenna with an open-circuit metasurface configuration achieves a bandwidth of 1110 MHz, ranging from 56.45 GHz to 57.56 GHz. In contrast, the antenna with a short-circuited metasurface configuration offers a bandwidth of 1020 MHz, ranging from 59.96 GHz to 60.98 GHz. The antennas in configuration 3 achieve dual bandwidth: the first ranges from 57.2 GHz to 58.36 GHz, and the second is between 60.11 GHz and 60.99 GHz. As shown in Figure 9, the coupling observed with S41, S42, or S43 is lower than −35 dB for all configurations.

**Figure 10:**
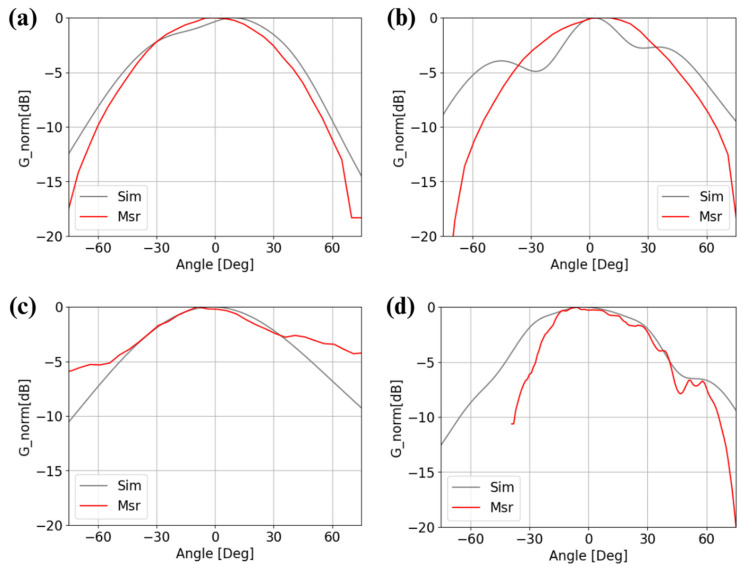
Radiation patterns. (**a**) E plane at 57.25 GHz in the OC metasurface configuration and (**b**) E plane at 60.5 GHz in the SC metasurface configuration; (**c**) H plane at 57.25 GHz in the OC metasurface configuration and (**d**) H plane at 60.5 GHz in the SC metasurface configuration.

The measured radiation patterns of the two configurations (OC and SC) in the E plane and H plane are shown in Figure 10. The measurements are in good agreement with the simulations. When comparing the simulations with the measurements, slight shifts can be observed. This discrepancy is due to the non-planarity of the BCB surface, caused by the presence of disks, rings, and vias at the metasurface interface, as well as some uncertainties in the exact values of permittivity and dielectric losses. It is also important to emphasize that the fabrication processes employed are not standardized, matured, or industrialized, as is the case for PCB technology. Instead, these processes have been meticulously adapted through iterative and labor-intensive procedures to achieve the current results, which are already promising. However, with further optimization and industrialization of the fabrication processes, we anticipate achieving significantly improved performance beyond the current outcomes.

## 6. Discussion

Several studies have demonstrated the potential of metasurface-based antennas to operate around 60 GHz, especially for WiGig and other high-data-rate applications. To this end, we have identified and listed several antennas operating around the 60 GHz frequency in order to compare their performance with that achieved by the proposed antenna, as summarized in Table 1. S. Ullah et al. [14] developed a 4 × 4 MIMO antenna on a 0.1 mm thick PCB substrate (Rogers, εr ≈ 2.9). The metasurface design, built with unit cells of 1.3 mm × 1.8 mm × 0.1 mm, allows for a wide frequency range of 57 to 63 GHz. This design achieved a gain of 8.2 dBi with good isolation levels reaching −54 dB. However, the size of the structure remains relatively large with 13 mm × 14 mm × 0.1 mm. Alassawi et al. [21] proposed both 2 × 2 and 4 × 4 MIMO-UWB antennas based on elliptic ring structures for 5G applications. Fabricated on Rogers 4003 (εr ≈ 3.55, thickness = 0.203 mm), the designs demonstrated good mutual coupling reduction through orthogonally arranged radiators. For the 2 × 2 configuration, the coupling achieved is −44 dB, while the 4 × 4 configuration reached −28 dB with the use of metallic isolators. Gains of 9.6 dBi (2 × 2) and 9.2 dBi (4 × 4) were achieved, demonstrating high efficiency for 5G communication systems. However, the dimensions of the 4 × 4 design remain significant (30 mm × 30 mm × 0.203 mm). Sharma et al. [22] introduced a compact 4 × 4 MIMO antenna operating at 60 GHz with dimensions of 16 × 16 × 0.254 mm^3^. This structure achieved a gain of 10.56 dBi while maintaining isolation better than −50 dB, making it highly competitive for mmWave applications. However, the design does not yet include reconfigurability, which limits its flexibility for adaptive systems. Moreover, the dimensions are sufficiently large to consider their integration into an RFIC chip. Furthermore, the technology used, namely PCB printing, implies that the antenna will be used outside the chip, leading to a greater number of interconnections, which will further result in additional losses and performance degradation.

The on-chip four-port MIMO antenna proposed here, including the advantages of a metasurface and of a BCB substrate, allows for achieving a more compact form factor and competitive performance. The overall size of our antenna is 7.54 × 7.54 × 0.055 mm^3^, which is significantly smaller than the aforementioned designs. Isolation between MIMO ports is maintained to be better than −35 dB, and gains ranging from 6.5 to 9 dBi are achieved depending on the operating frequency and on the metasurface configuration. Unlike previous works, our antenna is designed for frequency agility, with proof of concept presented here. Moreover, it should be noted that the integration of metasurfaces within this architecture further enhances the overall system performance by leveraging their unique properties for electromagnetic wave control. The roles of the metasurfaces used are well understood, but the main innovation does not lie in their application. Instead, we emphasize the complete integration of these technologies operating in the millimeter-wave band into an on-chip structure. This level of integration, achieved using cleanroom fabrication technologies commonly used in microelectronics, sets our work apart from most existing solutions in the literature, which primarily rely on PCB technologies. This approach reduces interconnections and thus losses, which constitutes a notable advancement. The future integration of diodes will enable dynamic reconfigurability, addressing a key limitation in existing designs.

This comparison demonstrates that our on-chip solution offers significant advantages in terms of miniaturization and reconfigurability while achieving competitive isolation and gain for mmWave applications.

## 7. Conclusions

In this work, we presented a 4 × 4 MIMO antenna integrated on a BenzoCycloButene (BCB) substrate and enhanced with a metasurface for high-performance operation at 60 GHz. With a compact volume of only 3.13 mm^3^, the antenna delivers an impressive gain of 8 dBi, with an average efficiency of 90%, and the frequency tuning range extends from 56.5 GHz to 61 GHz by simply reconfiguring the metasurface. The innovative manufacturing process enables seamless integration over active circuitry and provides the option to incorporate diodes for dynamic frequency matching. This design approach not only enables significant miniaturization but also opens the door to scaling down the technology for submillimeter wave applications, making it highly suitable for next-generation wireless systems, such as communication at 120 GHz and beyond.

## Figures and Tables

**Table 1:** Comparative study of the designed MIMO antenna with existing structures in the literature for 60 GHz applications.

Study	Technology	Dimensions (mm^3^)	Freq. (GHz)	Gain (dBi)	Isolation (dB)	Reconfigurability
[14]	PCB (Rogers, εr ≈ 2.9)	13 × 14 × 0.1 (2.6λ × 2.8λ × 0.02λ)	57.0–63.0	8.2	−54	No
[21]	PCB (Rogers 4003, εr ≈ 3.55)	30 × 30 × 0.203 (6.0λ × 6.0λ × 0.04λ)	58.0–63.0	9.6 (2 × 2), 9.2 (4 × 4)	−44 (2 × 2), −28 (4 × 4)	No
[22]	Rogers RT/Duroid 5880, εr = 2.2, h = 0.254 mm	16 × 16 × 0.254 (3.2λ × 3.2λ × 0.05λ)	58.925–60.66	10.56	<−50	No
Our work	On-chip, BCB substrate	7.54 × 7.54 × 0.055 (1.51λ × 1.51λ × 0.01λ)	57.0–63.0	6.5–9	<−35	Yes (proof of concept, OC/SC configurations)

## Data Availability

Data is contained within the article.

## Abbreviations

The following abbreviations are used in this manuscript:

BCB	BenzoCycloButene
MIMO	Multiple Input Multiple Output
AMC	Artificial Magnetic Conductor
SC	Short Circuit
OC	Open Circuit
ECC	Envelope Correlation Coefficient
DG	Diversity Gain
GSG	Ground–Signal–Ground
